# Tumor-specific radiosensitizing effect of the ATM inhibitor AZD0156 in melanoma cells with low toxicity to healthy fibroblasts

**DOI:** 10.1007/s00066-022-02009-x

**Published:** 2022-10-13

**Authors:** Julian Scheper, Laura S. Hildebrand, Eva-Maria Faulhaber, Lisa Deloch, Udo S. Gaipl, Julia Symank, Rainer Fietkau, Luitpold V. Distel, Markus Hecht, Tina Jost

**Affiliations:** 1 Department of Radiation Oncology, University Hospital Erlangen, Friedrich-Alexander-Universität Erlangen-Nürnberg, 91054 Erlangen, Germany; 2 Comprehensive Cancer Center Erlangen-EMN (CCC ER-EMN), 91054 Erlangen, Germany; 3 Translational Radiobiology, Department of Radiation Oncology, University Hospital Erlangen, Friedrich-Alexander-Universität Erlangen-Nürnberg, 91054 Erlangen, Germany

**Keywords:** Kinase inhibitors, Radiosensitivity, Healthy tissue, ATM, ATR

## Abstract

**Purpose:**

Despite new treatment options, melanoma continues to have an unfavorable prognosis. DNA damage response (DDR) inhibitors are a promising drug class, especially in combination with chemotherapy (CT) or radiotherapy (RT). Manipulating DNA damage repair during RT is an opportunity to exploit the genomic instability of cancer cells and may lead to radiosensitizing effects in tumors that could improve cancer therapy.

**Methods:**

A panel of melanoma-derived cell lines of different origin were used to investigate toxicity-related clonogenic survival, cell death, and cell cycle distribution after treatment with a kinase inhibitor (KI) against ATM (AZD0156) or ATR (VE-822, berzosertib), irradiation with 2 Gy, or a combination of KI plus ionizing radiation (IR). Two fibroblast cell lines generated from healthy skin tissue were used as controls.

**Results:**

Clonogenic survival indicated a clear radiosensitizing effect of the ATM inhibitor (ATMi) AZD0156 in all melanoma cells in a synergistic manner, but not in healthy tissue fibroblasts. In contrast, the ATR inhibitor (ATRi) VE-822 led to additive enhancement of IR-related toxicity in most of the melanoma cells. Both inhibitors mainly increased cell death induction in combination with IR. In healthy fibroblasts, VE-822 plus IR led to higher cell death rates compared to AZD0156. A significant G2/M block was particularly induced in cancer cells when combining AZD0156 with IR.

**Conclusion:**

ATMi, in contrast to ATRi, resulted in synergistic radiosensitization regarding colony formation in melanoma cancer cells, while healthy tissue fibroblasts were merely affected with respect to cell death induction. In connection with an increased number of melanoma cells in the G2/M phase after ATMi plus IR treatment, ATMi seems to be superior to ATRi in melanoma cancer cell treatments when combined with RT.

**Supplementary Information:**

The online version of this article (10.1007/s00066-022-02009-x) contains supplementary material, which is available to authorized users.

## Introduction

Melanoma is still a major health problem because of its high incidence, mortality rate, and few curative treatment options [1–4]. Recent achievements in research into biological mechanisms in melanoma have led to new potential treatment strategies [2, 3], e.g., immune therapies and targeting BRAF-related signal transduction pathways, and new understanding of regulatory proteins involved in DNA damage repair [1]. Initially, kinase inhibitors (KI) such as BRAF V600E-targeting vemurafenib and dabrafenib (both second generation) showed promising results in melanoma, and even more so when combined with IR [5]. Nevertheless, vemurafenib seems to lack adequate improvement of local or distant tumor control by the desired synergistic effects a simultaneous RT could offer [6, 7], but severe side effects were also published when combined with IR [8]. A current discovery to treat malignancies such as melanoma is offered by immunotherapy [9], and over 500 clinical trials are currently on going regarding melanoma and immunotherapy.

In managing melanoma, RT is a treatment option, especially in cases of bone or brain metastases [2]. The key mechanism of RT is induction of DNA double-strand breaks (DSB) [10]. Manipulating DNA damage repair whilst inducing DNA damage is an opportunity to exploit the genomic instability of cancer cells [11] and will probably lead to radiosensitization [12]. This is especially important in melanoma, which is known to have a low radiosensitivity [13]. Furthermore, there is clear evidence that cancer cells harbor mutations in their DNA damage repair pathways, especially in the pathway of homologous recombination (HR) as one of two main pathways of DSB repair [12]. This ineffective DSB repair can further be targeted with a combination of RT and DDR-inhibiting KI in cancer cells.

As there are plethora of kinases involved in DDR of HR and non-homologous end-joining (NHEJ) [1], further investigation is essential to increase the knowledge of cellular mechanisms of DNA repair and improve cancer therapy. Ataxia telangiectasia mutated (ATM) protein and ataxia telangiectasia and Rad3-related protein (ATR) are kinases related to DDR [14] that seem especially interesting when combined with RT. Targeting proteins involved in DDR pathways offers the potential of synergistic efficacy and is assumed to improve multimodal cancer treatment. Promising results of concomitant KI and RT have recently been published for melanoma and head and neck squamous cell carcinoma (HNSCC) and different targets, e.g., the previously mentioned ATM, ATR, DNA-PK, and PARP1/2 [12, 15–17].

As the inhibition of proteins of DDR pathways is gaining importance in various cancer treatments [18], we focused on melanoma cancer cells and inhibition of DDR through targeting the above-mentioned proteins ATR and ATM by using the ATM inhibitor (ATMi) AZD0156 and ATR inhibitor (ATRi) VE-822. Little is known regarding whether ATMi or ATRi is superior in terms of beneficial tumor cell targeting in combination with RT. The inhibitors used in our experiments are being investigated in several phase I–II trials (AZD0156: NCT02588105; VE-822: NCT02487095, NCT02589522). VE-822, also known as berzosertib, has already shown promising results in a first clinical phase II trial (*n* = 70) when combined with additional replication stress via chemotherapy for progression-free survival (PFS; berzosertib + gemcitabine vs. gemcitabine: 53.2 vs. 43 weeks) and median overall survival (OS; berzosertib + gemcitabine vs. gemcitabine: 22.9 vs 13 weeks) [19]. The radioresistance of melanoma is known to increase over time, especially in advanced stages when RT commonly takes place [20]. Combining DSB-inducing RT and inhibition of DSB repair mechanisms could potentially increase the therapeutic efficacy [21] by radiosensitization. This could improve tumor control in patients but may also increase side effects [22], as the surrounding healthy tissue is always affected by RT (i.e., the skin and other radiosensitive organs). We therefore focused our analyses not only on melanoma cancer cells, but also on healthy human fibroblasts.

## Materials and methods

### Cell culture, inhibitors, and irradiation

The experiments were performed in vitro using different melanoma cells and skin fibroblasts. ILSA and LIWE melanoma cells were derived from donors and were extracted from primary tumors of diseased patients at the Department of Dermatology of the University Hospital of Erlangen following approval by the institutional review board (ethics approval no. 204 17 BC). Accordingly, the human skin fibroblasts SBLF7 and SBLF9 were derived from healthy donors and extracted as described previously [23]. A375M, Mel624, and pMelL are low-passage cell lines derived from metastatic sites and purchasable melanoma cell lines provided by the Department of Immune Modulation at the University Hospital of Erlangen.

The human skin fibroblasts were grown in F‑12 (Gibco, Waltham, MA, USA) supplemented with 15% FBS (Merck, Darmstadt, Germany), 2% NEA (Merck, Darmstadt, Germany), and 1% penicillin/streptomycin (Gibco, Waltham, MA, USA). The medium used for the above-mentioned melanoma cells consisted of RPMI-1640 (Sigma Aldrich, München, Germany), supplemented with 20% FBS (Merck, Darmstadt, Germany), 1% NEA (Merck, Darmstadt, Germany), 1% pyruvate solution (Gibco, Waltham, MA, USA), 1% L‑glutamine (Merck, Darmstadt, Germany), 1% HEPES (Merck, Darmstadt, Germany), and 0.05% gentamicin (Merck, Darmstadt, Germany). ATM inhibitor AZD0156 and ATR inhibitor VE-822 were purchased from Selleckchem (Houston, TX, USA) and diluted with DMSO. This single dose per fraction of RT was set according to the normofractionation used in common clinical routine [24] and based on previously published work [12, 17, 25].

### Cell survival analyzed by colony-forming assay

A suitable number of cells was seeded using 3 mL of fresh medium in six-well plates. After 24 h, 5 nM and 10 nM of AZD0156 or 25 nM and 50 nM of VE-822 kinase inhibitor was added followed by irradiation with 2 Gy after an additional 3 h. Media change with drug-free medium was performed 24 h post treatment. Cells were incubated for 10 to 14 days. Cells were stained afterwards with methylene blue (#66725, Sigma Aldrich, München, Germany) for 30 min at room temperature. Colonies containing more than 50 cells were counted. Plating efficiency (1) and survival fraction (2) were calculated [26]. Survival curves for untreated and treated (AZD0156, VE-822) cells were plotted, and an additional radiation survival curve was generated after normalizing for the cytotoxicity induced by AZD0156 or VE-822 alone, to evaluate additive, antagonistic, or synergistic effects.

1 $$\mathrm{PE}\left(\text{plating efficiency}\right)=\frac{\mathrm{no}.\text{of colonies formed}}{\mathrm{no}.\text{of cells seeded}}\times 100{\%}$$

2 $$\mathrm{SF}\left(\text{survival fraction}\right)=\frac{\mathrm{no}.\text{of colonies formed after treatment}}{\mathrm{no}.\text{of cells seeded}\times \mathrm{PE}}$$

### Apoptosis and necrosis analysis

On day 0, around 25,000 to 50,000 Cells were seeded into T25 flasks with the aim of achieving a confluency of up to 60–80%. The cells were then incubated at 37 °C at 5% CO_2_ and kept under these conditions for 24 h. The medium was exchanged on day 1 for a serum-reduced medium (2% FBS) and the cells were divided into two groups: irradiated and non-irradiated. Afterwards, cells were treated with appropriate concentrations of kinase inhibitors for 48 h. After the first 3 ± 0.5 h of incubation, the group destined to be irradiated was then treated with 2 Gy RT using an ISOVOLT Titan X‑ray generator (GE, Ahrensburg, Germany). In order to avoid bias by DMSO-dissolved KI, an adequate volume of DMSO was added to untreated cells as a control. After an incubation of 48 h, the cells were harvested and then stained with annexin V‑APC (BD, Heidelberg, Germany) and 7‑amino-actinomycin D (7-AAD; BD, Heidelberg, Germany). After 30 min on ice, the cells were analyzed using a Cytoflex flow cytometer (Cytoflex, Beckman Coulter, Brea, CA, USA). The acquired data were analyzed with the Kaluza Analysis Software (Beckman Coulter, Brea, CA, USA). Annexin-positive/7-AAD-double-positive cells were considered “necrotic,” while annexin-positive/7-AAD-negative cells were defined as “apoptotic.” Cells with no staining were considered viable.

### Cell cycle analysis

The cells were processed as described in Sect. “Apoptosis and necrosis analysis,” harvested after 48 h, and fixed in 10 mL ethanol (70%) and 1 mL cell culture medium with 2% FBS for at least 24 h. The cells were then stained with Hoechst 33258 (Invitrogen, Eugene, OR, USA) and incubated on ice for 60 min. The cell cycle phases and their distribution were measured using flow cytometry. The analysis of the data was done with Kaluza Analysis Software.

### Immunostaining of RAD51 foci

Investigation of cells’ ability to undergo homologous recombination (HR) was performed as described previously [27]. Briefly, an adequate number of cells were seeded using cover slides and cultured to maximally 90% confluence. Culture medium was exchanged, and half of the samples were treated with 5 µM of DNA-PK and mTor inhibitor CC-115 to ensure a sufficient blockage of DNA-PK according to previous findings [28, 29]. After incubation of 24 h at 37 °C, cells were irradiated with a dose of 10 Gy by an ISOVOLT Titan X‑ray generator (GE, Ahrensburg, Germany). Slides were fixed and permeabilized with 4% formaldehyde and 0.1% Triton X‑100/PBS for 15 min 4 h post irradiation. Slides were blocked with 1% BSA overnight. Staining with primary antibodies—mouse anti-γH2AX (1:1500, Merck, Darmstadt, Germany) and rabbit anti-Rad51 (1:250, Abcam, Cambridge, UK)—was carried out overnight at 8 °C [30]. Slides were further stained with the secondary antibodies AlexaFluor488 goat anti-mouse and AlexaFluor594 chicken anti-rabbit (Invitrogen, Eugene, OR, USA). DAPI was applied for DNA staining (10236276001, Sigma Aldrich, St. Louis, MO, USA). Cover slides were mounted onto glass slides using Vectashield (Vector Laboratories, Burlingame, CA, US) and images were acquired by a Zeiss Imager Z2 fluorescence microscope (Zeiss, Oberkochen, Germany). Foci were quantified using Biomas Software (version V3.07/2012, MSAB, Erlangen, Germany).

### Statistics

The graphs presented in this work were created using Graph-Pad Prism 8 (GraphPad Software, San Diego, CA, USA). One-/two-tailed Mann–Whitney U test was used to analyze the data. A *p*-value ≤ 0.050 was determined as significant. Graphs were also generated using GraphPad Prism 8 software. Combination index (e.g., Bliss score) was calculated based on the Synergyfinder.org, a tool established by Tang et al. 2017 and Zheng et al. 2021 [31, 32]. Bliss score > 10 was defined as synergistic interaction.

## Results

In this study, five melanoma (A375M, ILSA, LIWE, Mel624, pMelL) and two healthy fibroblast (SBLF7, SBLF9) cell lines were treated with the ATMi AZD0156 or the ATRi VE-822 in combination with RT of 1 × 2 Gy. We first investigated the influence of concomitant KI on RT in colony-forming assays.

### Particularly ATMi has synergistic effects with RT in reducing clonogenic survival of melanoma cells

**Fig. 1:**
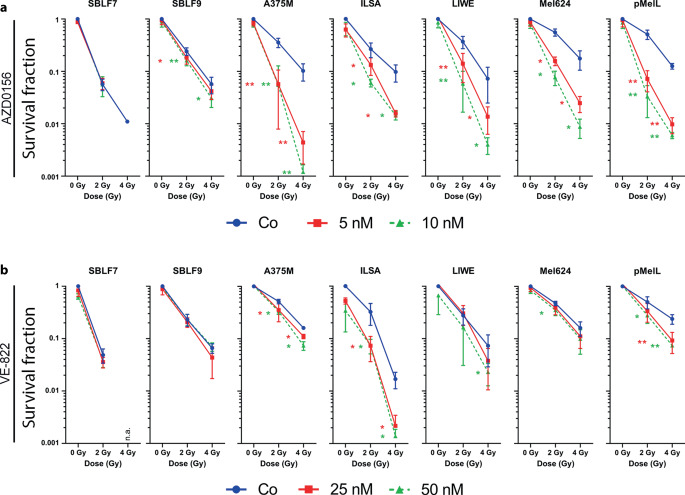
Cell survival after 10–14 days of incubation of melanoma cells and healthy fibroblasts under AZD0156 and VE-822 treatment (48 h) combined with RT. **a** Survival fractions of SBLF9, SBLF7, and A375M, ILSA, LIWE, Mel624, and pMelL under 5 nM or 10 nM ATMi (AZD0156) combined with a dose of 0 Gy, 2 Gy, or 4 Gy. **b** Survival fractions of SBLF9, SBLF7, and A375M, ILSA, LIWE, Mel624, and pMelL under 25 nM or 50 nM ATRi (VE-822) combined with a dose of 0 Gy, 2 Gy, or 4 Gy. To the control (*Co*; *blue line*), an equivalent volume of DMSO was added. No colonies of SBLF7 were measurable after AZD0156 + RT and VE-822 + RT. Each value represents mean ± SD (*n* = 4). Significance (RT vs. RT + 25 nM, *red line*; RT vs. RT + 50 nM, *green line*) was determined by one-tailed Mann–Whitney U test; *p ≤ 0.050 and **p ≤ 0.010

We first performed colony-forming assays to measure effects of simultaneous KI and RT treatment. Two healthy fibroblast cell lines and five melanoma cell lines (A375M, ILSA, LIWE, Mel624, and pMelL) were used for the analyses (Fig. 1). Cells were treated with concentrations of 5 or 10 nM of ATMi AZD0156 (Fig. 1a) and 25 nM or 50 nM of ATRi VE-822 (Fig. 1b) according to our previously published work [12].

AZD0156 + RT treatment led to either no or only slight reduction of cell survival compared to irradiation alone in fibroblasts representing healthy tissue. In melanoma cells, combination treatment of 5 nM and 10 nM + 2 or 4 Gy led to a clear reduction of the survival fraction in all tested cancer cell lines. Additionally, the survival fraction was normalized to AZD0156 treatment alone to discover possible additive or synergistic effects (Figure S1, A). In all melanoma cell lines except ILSA, the normalized data were significant when comparing to irradiation, but not in healthy fibroblasts.

The cell lines were also tested with ATRi VE-822 in combination with irradiation of 2 or 4 Gy (Fig. 1b). Survival fraction decreased in SBLF7 and SBLF9 in an RT-dependent manner. Additional KI therapy did not lead to an increase of this effect. Mel624 and pMelL responded to 50 nM + 2 Gy treatment compared to 2 Gy significantly (*p* = 0.029, *p* = 0.016), whereas A375M and ILSA showed reduction of survival fraction even after 25 nM + RT (*p* = 0.050). LIWE responded to radiation, but no increased effect was detectable during combination therapy of 25 and 50 nM + 2 Gy and 25 nM + 4 Gy. After normalization of our data (supplementary Figure S1, B) to KI treatment alone, only A375M showed a significant reduction of cell survival (*p* ≤ 0.050).

Based on our findings in the colony-forming assay, we additionally calculated the Bliss score to test for synergistic or additive effects [31, 32]. Treatment of cells with AZD0156 (ATMi) and RT lead to a synergistic enhancement of toxicity (Bliss score > 10; marked in green) in tumor cells but not in healthy fibroblasts and cancer cell line ILSA (Tables 1 and 2). VE-822 in combination with IR showed additive enhancement in healthy fibroblasts, ILSA, LIWE, and Mel624. Synergistic interactions occurred only in A375M and pMelL cells (marked in green; statistics: supplementary Tables S1 and S2).

**Table 1:** Synergy score summary table of cell lines treated with AZD0156 (ATMi)

Cell line	ZIP	Loewe	HSA	Bliss
SBLF7	−0.3521	−0.1754	−0.1169	−0.5161
SBLF9	2.1001	3.8063	3.8362	2.1001
A375M	*14.5542*	*20.0601*	*20.0851*	*15.5875*
ILSA	6.6065	10.8123	10.8411	4.7611
LIWE	*16.6344*	*17.9149*	*17.9394*	*17.0067*
Mel624	*23.5393*	*28.8961*	*28.9135*	*23.0875*
pMelL	*25.5602*	*30.0452*	*30.0646*	*27.1163*

*ZIP* Zero Interaction Potency Model, *Loewe* Loewe additivity Model, *HSA* Highest single agent Model, *Bliss* Bliss independence Model

Italicized values represents scores defined as “synergistic” based on the underlying models

**Table 2:** Synergy score summary table of cell lines treated with VE-822 (ATRi)

Cell line	ZIP	Loewe	HSA	Bliss
SBLF7	−0.3838	0.9488	1.0004	−0.2166
SBLF9	−0.2061	0.7955	0.8398	−0.5355
A375M	*11.5447*	*11.3848*	*11.4113*	*12.7829*
ILSA	2.0473	16.7183	16.8401	5.1468
LIWE	0.9050	2.9709	3.0129	0.3846
Mel624	3.6565	7.7842	7.8123	4.1345
pMelL	*14.7453*	*14.5147*	*14.5421*	*16.4310*

### Increased ATRi-driven toxicity in melanoma cells and healthy fibroblasts

**Fig. 2:**
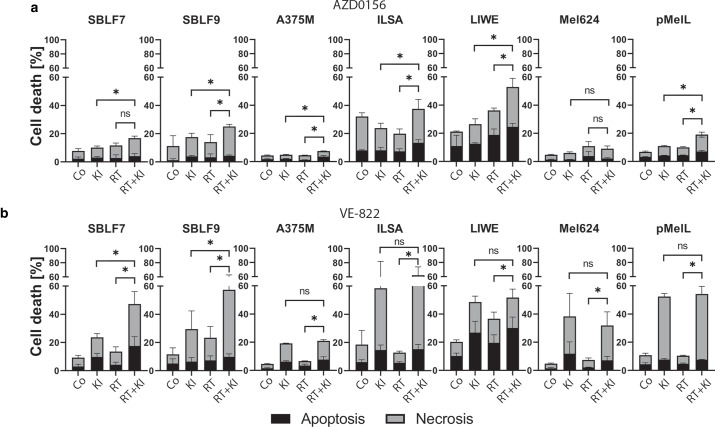
Influence of AZD0156 and VE-822 combined with RT on cell death. Cell death analysis was performed by flowcytometry and annexin V and 7‑AAD staining to detect apoptosis (annexin V) and necrosis (7-AAD) in two fibroblast cell lines and five skin cancer cell lines. Cells were treated for 48 h with either 1 µM of kinase inhibitor (*KI*), 2 Gy (*RT*), or a combination of 2 Gy and 1 µM of kinase inhibitor (*RT* *+* *KI*). For the control (*Co*), DMSO was used corresponding to the volume of KI. **a** Cells were treated with 1 µM ATM inhibitor AZD0156. **b** Cells were treated with 1 µM ATR inhibitor VE-822. Each value represents mean ± SD of combined cell death (apoptosis + necrosis; *n* = 4). Significance of KI vs. RT + KI and RT vs. RT + KI was determined by two-tailed Mann–Whitney U test; *p ≤ 0.050

To gain more knowledge on tumor cell killing properties of simultaneous KI and RT treatment, we then investigated cell death by flow cytometric analysis of apoptosis and necrosis. ATMi or ATRi was applied at a concentration of 1 µM, since preliminary data at concentrations used in colony-forming experiments did not show cellular toxicity (data not shown). Furthermore, adaption to concentrations reached by oral treatment of patients took place [33, 34]. Annexin V-APC and 7‑AAD were used to stain and distinguish viable, apoptotic, and necrotic cells using flow cytometry (Fig. 2; gating strategy: supplementary Figure S2).

Four out of five melanoma cell lines (A375M, ILSA, LIWE, and pMelL; Fig. 2a) treated with a combination of RT and KI AZD0156 had a significantly higher percentage (*p* ≤ 0.050) of cell death compared to the irradiated group. Additionally, the skin fibroblasts SBLF9 also showed a significant increase in cell death (*p* ≤ 0.050). Furthermore, the comparison of AZD0156 treatment to the combination of KI + RT also showed a significant increase of cell death in the same four melanoma cell lines, but also in both healthy fibroblast cell lines (*p* ≤ 0.050), albeit here to a lesser extent. Melanoma cells responded variously to the treatment, e.g., with a minimum cell death value (RT + KI) of 7.5 ± 0.8% in A375M and maximum cell death value of 52.8 ± 6.9% in LIWE cells.

Noticeably, combined treatment of ATR inhibitor (ATRi) VE-822 and RT showed a high and significant increase in cell death compared to RT in both fibroblast cell lines SBLF9 and SBLF7. Regarding the melanoma cells, ATRi alone was a strong cell death inducer and addition of RT did not further significantly increase this (Fig. 2b; *p*-value ≤ 0.050). These data are supported by the analysis of the sub-G1 phase, representing cells under DNA fragmentation as an indicator of apoptosis (Figure S3).

### AZD0156 in combination with RT results in higher G2/M arrest compared to single treatments

**Fig. 3:**
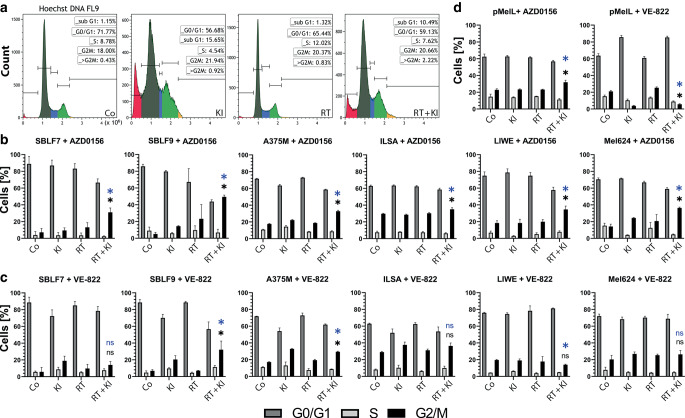
Cell cycle analysis of cells under KI treatment, RT, or a combination of both based on flow cytometry and Hoechst 33342 staining. For the control (*Co*), DMSO was used corresponding to the volume of kinase inhibitor. **a** Representative histograms of gating strategy. Mel624 cells were treated for 48 h with AZD0156, RT, or AZD0156+RT. **b** Healthy fibroblasts (SBLF9, SBLF7) and skin cancer cells (A375M, ILSA, LIWE, Mel624) were treated with ATM inhibitor AZD0156 and the proportion of cells in cell cycle phases G0/G1, S, and G2/M was measured. **c** Cells treated with ATR inhibitor VE-822. **d** pMelL treated with AZD0156 and VE-822. For the control (*Co*), DMSO was used corresponding to the volume of KI. Each value represents mean ± SD (*n* = 4). Significance regarding the influence of KI on RT (blue *: KI vs. RT + KI and black *: RT vs. RT + KI) was determined by Mann–Whitney U test for the G2/M phase; *p ≤ 0.050

Cellular sensibility for irradiation changes with the cell cycle phases and cells are most sensitive in G2/M [35]. In order to investigate possible underlying explanations for the different responses to ATMi or ATRi treatment, we measured the cell cycle distribution by DNA staining (Fig. 3). The histograms shown in Fig. 3a represent the evaluation process and gating strategy of our study. Analysis of the cell cycle distribution was done with flow cytometry and Hoechst 33258 staining. We were particularly interested in G2/M phase arrest, as here, sensitivity to IR is higher than in other cell cycle phases [36].

After combination of RT with AZD0156, all of the investigated melanoma cell lines (Fig. 3b, d) showed a significant increase of cells in G2/M (*p* ≤ 0.050) in comparison to the single RT or KI treatment. Additionally, both healthy fibroblast cell lines (SBLF7 and SBLF9) showed a similar significant increase in cells in G2/M (*p* ≤ 0.050). After treatment with VE-822, the population of cells in G2/M phase increased significantly only for fibroblasts SBLF9 (*p* = 0.036) and melanoma cell line A375M (*p* ≤ 0.050) compared to RT or KI treatment alone. Interestingly, VE-822 leads to a significant reduction of the cell population in G2/M after combination of KI with RT compared to RT treatment alone in pMelL cells, but to a slight increase compared to KI alone (Fig. 3d).

### Status of homologous recombination efficiency in melanoma and fibroblast cell lines

**Fig. 4:**
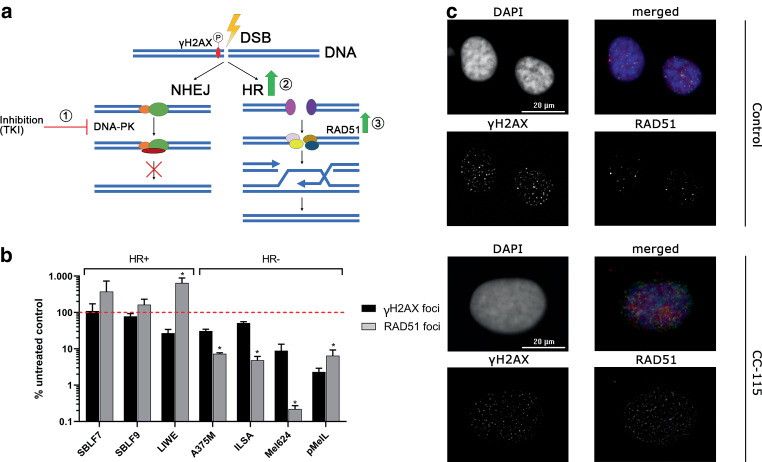
Capability of melanoma and fibroblast cell lines to undergo essential DNA damage repair by homologous recombination. Irradiation could lead to DSB, marked by phosphorylation of histone H2AX. **a** DSB can be repaired via NHEJ or HR. The NHEJ pathway was blocked by inhibition of the central protein DNA-PK, an essential protein in the signal cascade of NHEJ, by treating the cells with CC-115 (①). Since there is evidence that tumor cell lines often harbor mutations in the HR pathway, we forced them to use this repair pathway by treating the cell with a DNA-PK inhibitor. After irradiation (1 × 10 Gy), cells are forced to use the alternative pathway HR (②) associated with RAD51, a key player in HR. Upregulation of RAD51 (③), while forcing cells to HR, can be used to determine HR efficiency. Stable or decreasing RAD51 expression suggests an HR deficiency. **b** Analysis of RAD51 (*grey bars*) and yH2AX (*black bars*) foci of SBLF9, SLBLF7 (healthy control) and five melanoma cell lines after 10 Gy irradiation and treatment of cells with the DNA-PK inhibitor for 48 h. Untreated samples were set as 100%, as represented by the *red dashed* line. Cells with an increasing number of RAD51 foci after blocking NHEJ were defined as HR proficient (SBLF7, SBLF9, and LIWE), whereas cells with a decreasing number were defined as HR deficient (ILSA, A375M, Mel624, and pMelL). Each value represents mean ± SD (*n* = 3). Significance was determined by one-tailed Mann–Whitney U test; *p ≤ 0.050. **c** Representative microscope images of melanoma cell line LIWE without treatment and after blockade of NHEJ via DNA-PKi CC-115. Cells were stained with DAPI (nucleus) and primary antibodies mouse anti-γH2AX (1:1500, Merck, Darmstadt, Germany) and rabbit anti-Rad51 (1:250, Abcam, Cambridge, UK)

As irradiation leads to DNA damage, we investigated the potential of the cell lines for HR (Fig. 4a) for a better understanding of underlying cellular mechanisms. ATRi showed stronger KI-related toxicity but colony forming was decreased by ATMi more distinctly. An explanation might be given by intrinsic deficiencies in the DNA damage response, e.g., HR.

RAD51 staining (Fig. 4b) showed that healthy fibroblasts (supplementary Figure S4) raise the number of RAD51 foci after blocking the NHEJ-related protein DNA-PK, assuming a proficient HR pathway, as well as in the BRAF wildtype melanoma cell line LIWE. Cancer cell lines A375M, ILSA, Mel624, and pMelL showed a reduction of RAD51 foci after blocking the second DSB non-homologous end-joining repair pathway by treating cells with a DNA-PK inhibitor. These results suggest ineffectiveness in the HR pathway for the mentioned cell lines [30].

## Discussion

RT is frequently applied as a part of multimodal management of advanced melanoma [37, 38] and its biological efficacy is mainly based on induction of DNA DSBs [12]. DSBs activate different cellular processes including cell cycle arrest, repair mechanisms, cell death, or senescence [39]. There are two dominant forms of DSB repair mechanisms, namely HR and NHEJ [40], which are both suggested to be p53 dependent. This is ultimately related to the activity of the proteins ATM and/or ATR [41, 42]. As healthy cells do not have the same restricted DNA repair capacity typically found in cancer cells [43], we suggested that healthy cells—fibroblasts in our model system—should adapt better to inhibition of DDR, while targeting ATR or ATM with specific inhibitors. To analyze this, colony-forming assays of fibroblasts and melanoma tumor cells were performed. As removing cancer cells from a reproductive cycle is essential for successful tumor treatment, recent studies have suggested that cell cycle arrest leading to, e.g., senescence, is a relevant mechanism in tumor treatment [17, 44]. Our results show that the ATMi AZD0156 lowers the survival rate significantly in every melanoma cell line tested, while this was hardly observed in healthy fibroblasts. In contrast, colony forming was less inhibited by the combination of RT and ATRi compared to RT + ATMi.

In order to identify additive or synergistic mechanisms of the combination therapy, we normalized the colony-forming data. We observed a synergistic effect of ATMi + RT (Figure S1) for four out of five tested melanoma cell lines, but only one case of synergistically decreased survival after ATRi + RT. This leads to the assumption of enhancing local tumor control by combining RT and AZD0156 treatment without an increase in healthy tissue damage. Our assumption is supported by calculation of the Bliss score of the cell survival data, which shows particularly significant synergisms between RT and ATM inhibition with AZD0156 in most of the melanoma cell lines, but not in healthy cells (Tables 1 and 2). Since healthy tissue is always involved in RT, especially when irradiation takes place locally to radiosensitive organs (i.e., gland or swallowing apparatus), increasing killing of tumor cells while sparing non-malignant tissue is of particular interest in the field of radiation oncology. ATRi and ATMi showed different outcomes, which might be based on the different target proteins and their modes of action at the molecular level regarding cell death and cell cycle regulation. Moreover, normal tissue cells respond differently to the multimodal treatment compared to tumor cells. This finding is not surprising, as several survival and especially repair mechanisms are based on mutations in cell cycle regulation or DNA repair-related proteins, being a central part of the suggested “hallmarks of cancer” [45].

Regarding tumor cell death induction, VE-822 plus IR led to higher cell death rates compared to AZD0156 in healthy fibroblasts. In general, the five tested cancer cell lines showed heterogeneous cell death responses, especially to ATMi treatment. We assume that the mode of action is different between ATR and ATM inhibition, assuming the establishment of short-term toxicity through ATRi and long-term effects by ATMi, leading to reduced clonogenicity. This might be driven by dysregulation of the cell cycle differently by the different inhibitors. This explanation is also supported by Menon and Povirk, who suggested that ATM is primarily involved in detecting and binding to DSBs, resulting in activation and adaption of cell cycle checkpoints and DNA repair pathways, respectively [46]. All cancer cell lines responded strongly to ATRi but without leading to a synergistic interaction with RT in colony formation. Based on the longer incubation time of cell death analysis, one should take off-target effects into consideration. Currently, there is no published evidence for off-target binding of ATRi VE-822 or ATMi AZD0156.

In order to increase the efficiency of RT, one should also take into consideration that cells in different cell cycle phases have varying susceptibility to IR [36]. The G2/M phase is of special interest: IR induces cell cycle arrest at G2 and effects are strongest therein [47, 48]. ATM and ATR are suggested to induce cell cycle arrest in the G2 phase [49]. Inhibition of either kinase could therefore be detrimental to our original intent. Our experiments show that the tested cell lines treated with ATMi and RT induce G2 cell cycle arrest more often than cell lines treated with ATRi and RT in healthy and cancer cells. There is evidence that G2 cell cycle blockage might lead to senescence based on the discovery that p21 is involved in G2/M checkpoint regulation, too [50]. This also correlates with our findings of colony-forming inhibition by AZD0156. Since ATM plays a central role in DDR and ATR is more related to DNA replication, this mechanism may avert cells from reaching the G2 phase after ATRi. This is consistent with findings of Li and colleagues showing an increased expression of p‑ATM after irradiation [51]. Based on our findings, we claim that ATRi primarily leads to short-term effects such as cell death [52]. In contrast, ATMi is evidently connected with long-term effects, e.g., senescence [53]. Based on our data, both inhibitors might use different mechanisms of action, which is important to keep in mind, as the cellular response (e.g., apoptosis, necrosis) affects treatment outcome. Meaning, improvement of therapy is also influenced by the immune response (immunogenic cell death) [54] and immune modulation [55], or failure of cell death by cells reaching a senescence status [56]. This is also connected to the ability of DNA damage repair, which is often dysregulated in tumor cells.

Insufficient DNA repair might lead to a strong increase in the toxicity of DDR inhibitors. Therefore, we focused on the ability of the tested cell lines to use HR for DNA damage repair in vitro, influencing the susceptibility of our cells to ATRi or ATMi. Four out of five melanoma cell lines were found to harbor an inefficient HR pathway, while healthy fibroblasts and one melanoma cell line (LIWE) were presumed to use HR adequately. LIWE and healthy fibroblasts showed no reduced clonogenicity following treatment with RT and ATRi.

We assume that cells facing an ineffective HR pathway respond better to ATM than to ATR inhibition. Regarding the dominant toxicity of ATRi, it is obvious that different modes of action beside HR influence the outcome of KI and combinatory treatment with RT. Further research will be necessary, e.g., RT-PCR analysis of central proteins related to cellular processes of apoptosis, autophagy, and others, as controversial observations have been published [57, 58]. Diverse treatment-related outcome could be additionally based on patient-specific individual radiosensitivity [45, 59]. Noticeably, the melanoma cell line LIWE responded strongly to VE-822 treatment but did not lead to an increase of cells in the sub-G1 phase in contrast to ILSA. Translocation of phosphatidylserine is connected to an early stage of apoptosis, while DNA fragmentation is characteristic of late-phase apoptosis. We suggest, because of underlying patient-specific mutations, that VE-822 leads to different intensities of toxicity and less death. Furthermore, this effect on melanoma cells treated with different inhibitors of the DDR was observed in other studies regarding targeted therapy using PARP1/2 and DNA-PK inhibitors [15, 52, 60].

A limitation of our study is the fact that mutation profiles of our skin-derived cell lines are currently not available. The functional assessment of HR status alone seems not to be an appropriate marker for treatment response to AZD0156 or VE-822, since HR status and KI susceptibility do not confirm each other. Further investigation of the used cell lines, including mutation profiles, should be conducted, as the used HR assay and the observation of G2/M induction allow explanation of only parts of the results. However, there is great evidence that intervening in DNA repair (including proteins like ATM, ATR, PARP1/2, BRCA1/2) affects the outcome of RT in a beneficial manner in melanoma cancer cells. Moreover, the *TP53* status seems to influence the outcome of RT in ATM-mutated patients [46]. Therefore, the status of these above-mentioned players should be investigated in the future. Additionally, cell lines resistant to the investigated kinase inhibitors should be used in follow-up projects to gain a deeper understanding of cellular processes, and experiments going ahead from the in vitro level to ex vivo or in vivo will be important, according to our previously published data of radiosensitization in blood samples from cancer patients under KI therapy [61]. Concerning our study with melanoma cells, ATMi showed especially promising results in combination with RT and should thus be further investigated. Similar results have recently been published in other studies analyzing interactions in, e.g., HNSCC and melanoma cells [13, 15, 17, 52]. Furthermore, one aspect should be taken into account in the future, since previously published data postulated an elevated level of ATM expression in several cancer cells. In contrast to our data, Moschos et al. and Hussain et al. suggested an increase in radioresistance based on a thorough ATM-related DNA repair. Further analysis should be done to check the role of ATM in the radiation response of cancer and non-malignant cell lines [20, 62].

## Conclusion

ATM inhibition led to efficient radiosensitization during colony forming in melanoma cells, while healthy tissue fibroblasts were merely affected in a RT-related manner. This differing effect in tumor and healthy tissue suggests ATMi to be promising combination drug with IR for future clinical trials. Nevertheless, interindividual differences must be considered in this evaluation, as the responses vary in both healthy and cancer cells.

### Supplementary Information

The supplementary information includes normalized data of cell survival of melanoma cell lines and healthy fibroblasts under AZD0156 and VE-822 treatment combined with RT, the gating strategy of our cell death analysis, analysis of cells in sub-G1 phase based on flow cytometry measurement and additional representative microscope images of the analysis of RAD51 and yH2AX.
