# Supplementary material for: Tumor-specific radiosensitizing effect of the ATM inhibitor AZD0156 in melanoma cells with low toxicity to healthy fibroblasts
Source: Strahlenther Onkol. 2022 Oct 13;199(12):1128–39. doi: 10.1007/s00066-022-02009-x (PMC10673781; doi:10.1007/s00066-022-02009-x)
Supplement: Supplementary file 1 — The supplementary information includes normalized data of cell survival of melanoma cell lines and healthy fibroblasts under AZD0156 and VE-822 treatment combined with RT, the gating strategy of our cell death analysis, analysis of cells in sub-G1 phase based on flow cytometry measurement and additional representative microscope images of the analysis of RAD51 and yH2AX. [file 66_2022_2009_MOESM1_ESM.docx]

Supplementary Material

**
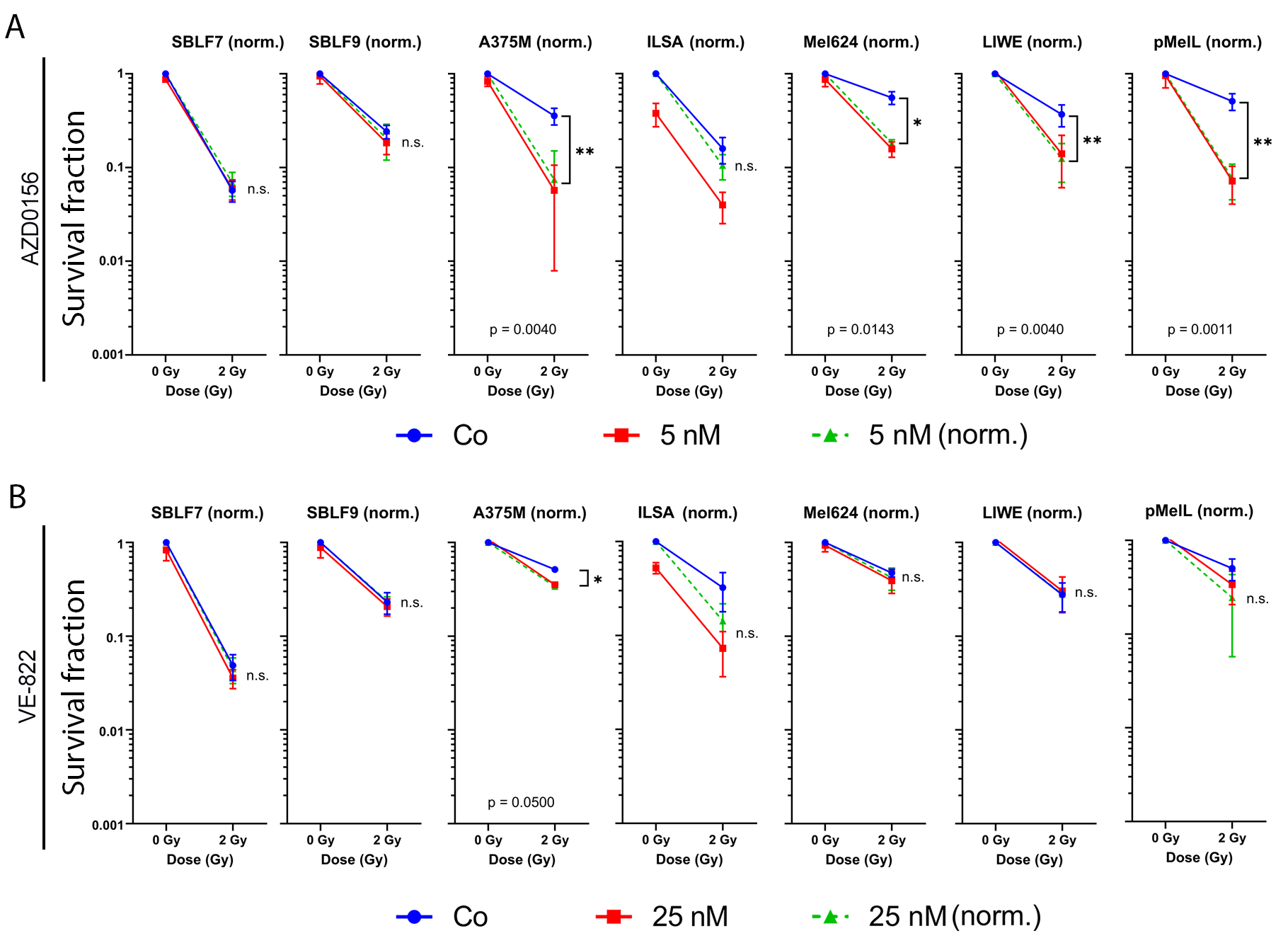
Figure S1**: Normalized data of cell survival of melanoma cell lines and healthy fibroblasts under AZD0156 and VE-822 treatment combined with RT. Survival fractions of SBLF9, SBLF7 and Mel624, A375M, pMelL and LIWE under 5 nM ATMi (AZD0156) (A) combined with either a dose of 0 Gy or 2 Gy. The 5 nM AZD0156 inhibitor treated curve was shifted parallel to the control curve to depict an interaction (dashed line). Survival fractions of SBLF9, SBLF7 and Mel624, A375M, pMelL and LIWE under 25 nM ATRi (VE-822) (B) combined with either a dose of 0 Gy or 2 Gy. The 25 nM VE-822 inhibitor treated curve was shifted parallel to the control curve to visualize an interaction (dashed line). Each value represents mean ± SD (n = 4). Significance was determined by two-tailed Mann-Whitney-U test * p ≤ 0.050 and ** p ≤ 0.010.

**Table S1**: Synergy Score Statistic Table of cell lines treated with AZD0156 (ATMi)

| Cell line | ZIP p-Value | Loewe p-Value | HSA p-Value | Bliss p-Value |
| --- | --- | --- | --- | --- |
| SBLF7 | 1.37e-01 | 5.00e-01 | 6.52e-01 | 1.77e-02 |
| SBLF9 | 5.86e-11 | 3.39e-20 | 1.71e-20 | 5.86e-11 |
| A375M | 2.06e-11 | 1.48e-114 | 6.24e-115 | 6.74e-82 |
| ILSA | 1.41e-12 | 2.94e-28 | 2.01e-28 | 3.26e-15 |
| LIWE | 5.14e-72 | 1.72e-73 | 9.04e-74 | 7.22e-61 |
| Mel624 | 2.68e-91 | 2.34e-153 | 1.07e-153 | 2.48e-123 |
| pMelL | 1.33e-21 | < 2e-324 | < 2e-324 | 4.46e-293 |

**Table S2**: Synergy Score Statistic Table of cell lines treated with VE-822 (ATRi)

| Cell line | ZIP p-Value | Loewe p-Value | HSA p-Value | Bliss p-Value |
| --- | --- | --- | --- | --- |
| SBLF7 | 3.45e-01 | 2.00e-02 | 1.41e-02 | 6.46e-01 |
| SBLF9 | 9.16e-01 | 7.57e-01 | 7.43e-01 | 8.24e-01 |
| A375M | 6.56e-07 | 3.77e-04 | 3.64e-04 | 2.08e-04 |
| ILSA | 2.26e-01 | 5.91e-06 | 3.60e-06 | 1.82e-02 |
| LIWE | 8.24e-01 | 4.11e-01 | 4.03e-01 | 9.34e-01 |
| Mel624 | 6.38e-02 | 3.93e-05 | 3.67e-05 | 8.73e-02 |
| pMelL | 3.89e-03 | 2.69e-04 | 2.60e-04 | 6.70e-04 |


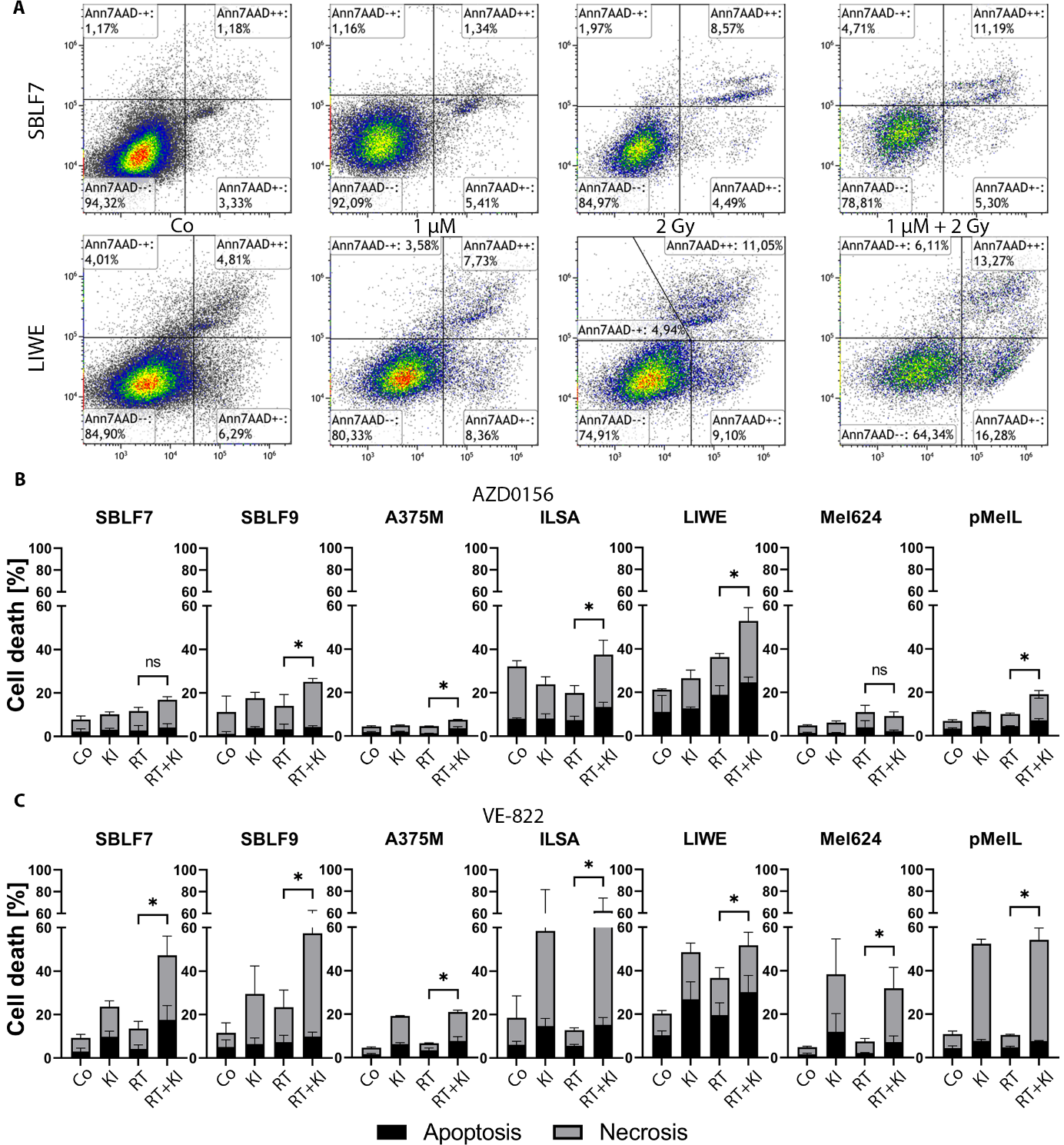


**Figure S2**: Gating strategy of Annexin V (apoptosis) and 7-AAD (necrosis) staining of healthy fibroblasts SBLF7 and skin cancer cell line LIWE under treatment with 1 µM AZD0156 w/o RT. Ann7AAD --: alive, Ann7AAD +-: apoptotic, Ann7AAD ++: necrotic cells.

| 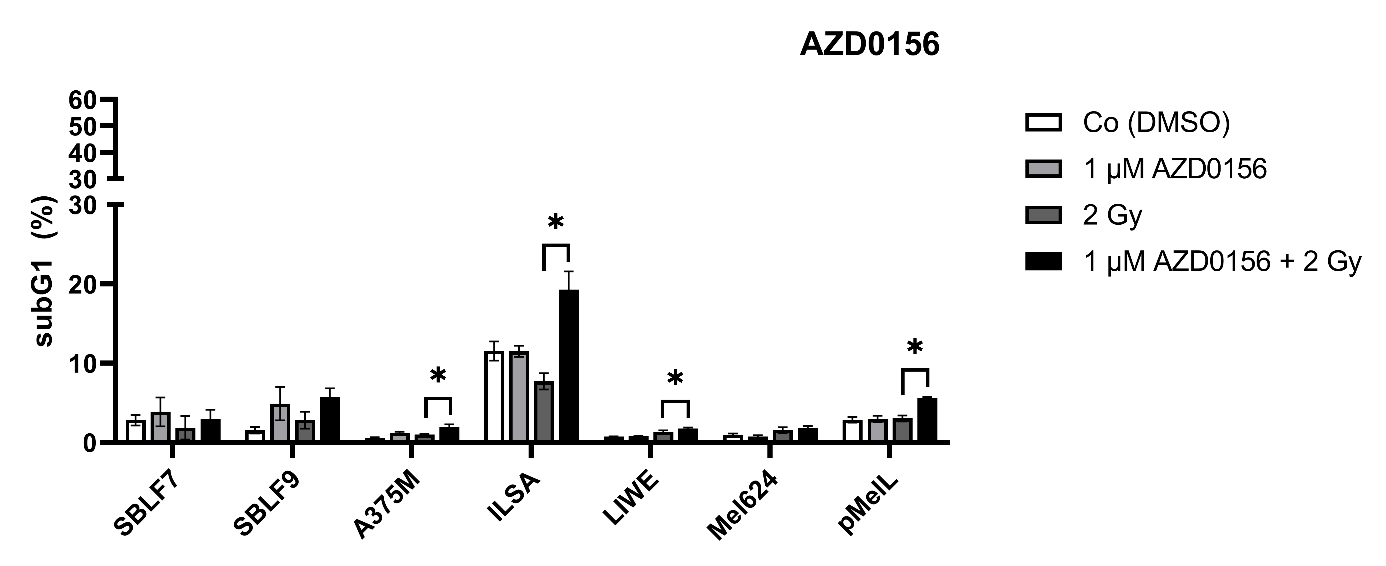  A |
| --- |
| 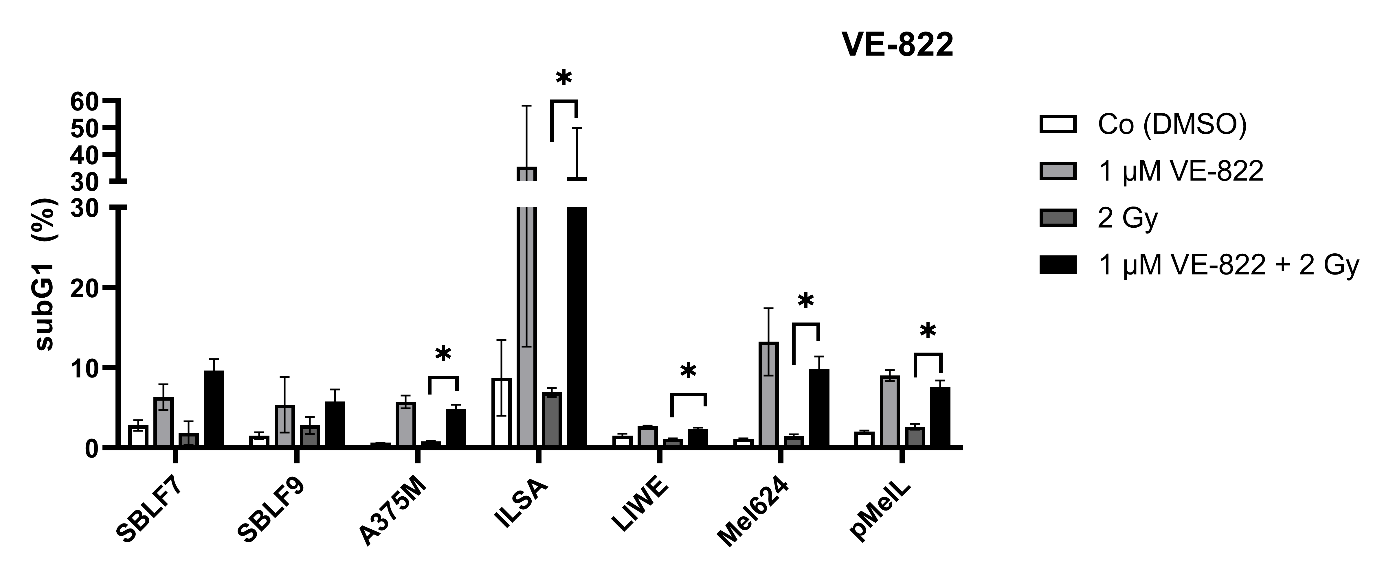  B |
| **Figure S3**: Analysis of cells in sub-G1 phase based on flow cytometry measurement of Hoechst 33342 staining. (A) Cells treated with ATM inhibitor AZD0156 for 48 h, a 2 Gy dose or the combination of AZD0156 and 2 Gy. To the control the equivalent volume of DMSO was added. (B) Cells treated with ATR inhibitor VE-822 for 48 h, a 2 Gy dose or the combination of AZD0156 and 2 Gy. To the control the equivalent volume of DMSO was added. Each value represents mean ± SD (n = 4). Significance was determined by Mann-Whitney-U test * p ≤ 0.050. |


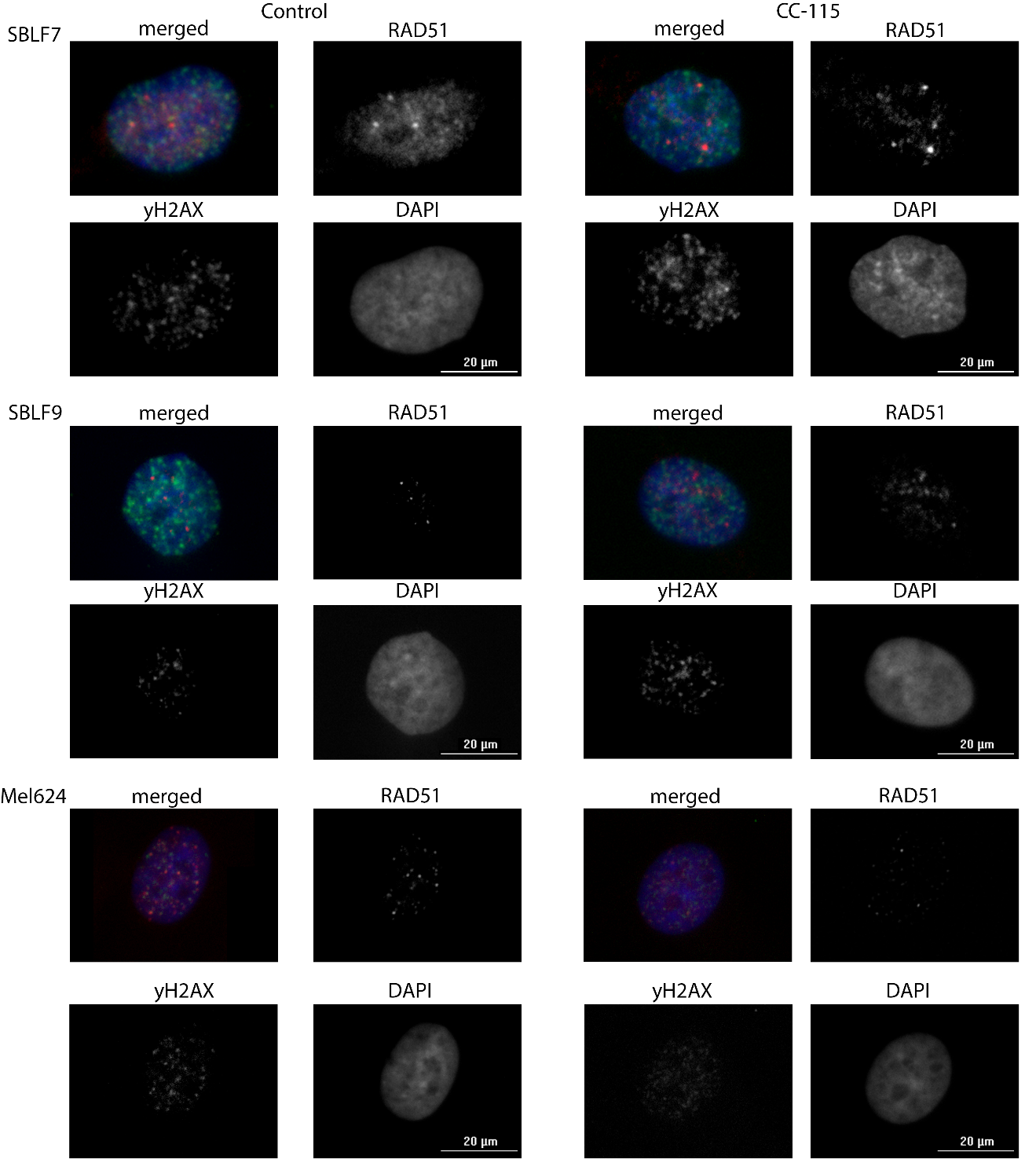


Figure S4: Representative microscope images of the analysis of RAD51 (red) and yH2AX (green) foci of SBLF7, SLBLF9 (healthy control) and melanoma cell line Mel624 after 10 Gy-irradiation and treatment of cells after blockade of NHEJ via DNA-PKi CC-115 for 48 h. Cells were stained with DAPI (nucleus) and primary antibodies mouse anti-γH2AX (1:1500, Merck, Darmstadt, Germany) and rabbit anti-Rad51 (1:250, abcam, Cambridge, UK). Scale = 20 µm.
